# Distinct Regulatory Genomic Architectures Distinguish Early-Onset from Late-Onset Alzheimer’s Disease

**DOI:** 10.3390/genes17020186

**Published:** 2026-01-31

**Authors:** Iliannis Yisel Roa-Bruzón, Celeste Patricia Gazcón-Rivas, Asbiel Felipe Garibaldi-Ríos, Luis Félix Duany-Almira, Martha Patricia Gallegos-Arreola, Claudia Azucena Palafox-Sánchez, Daniel Ortuño-Sahagún, Luis Eduardo Figuera, Manuel Alejandro Rico-Méndez, Yeminia Valle

**Affiliations:** 1 Doctorado en Genética Humana, Centro Universitario de Ciencias de la Salud, Departamento de Biología Molecular y Genómica, Universidad de Guadalajara, Guadalajara 44340, Jal, Mexico; iliannis.roa8556@alumnos.udg.mx (I.Y.R.-B.); celeste.gazcon1117@alumnos.udg.mx (C.P.G.-R.); asbiel.garibaldi4757@alumnos.udg.mx (A.F.G.-R.); luis.duany8455@alumnos.udg.mx (L.F.D.-A.); manuel.rico8557@alumnos.udg.mx (M.A.R.-M.); 2 Instituto de Investigación en Ciencias Biomédicas, Centro Universitario de Ciencias de la Salud, Departamento de Clínicas Médicas, Universidad de Guadalajara, Guadalajara 44340, Jal, Mexico; claudia.palafox@academicos.udg.mx; 3 Laboratorio de Neuroinmunobiología Molecular, Instituto de Neurociencias Translacionales, Centro Universitario de Ciencias de la Salud (CUCS), Universidad de Guadalajara, Guadalajara 44340, Jal, Mexico; daniel.ortuno@academicos.udg.mx; 4 División de Genética, Centro de Investigación Biomédica de Occidente (CIBO), Centro Médico Nacional de Occidente (CMNO), Instituto Mexicano del Seguro Social (IMSS), Guadalajara 44340, Jal, Mexico; marthapatriciagallegos08@gmail.com (M.P.G.-A.); luis.figuera@academicos.udg.mx (L.E.F.); 5 Instituto de Genética Humana “Dr. Enrique Corona Rivera”, Departamento de Biología Molecular y Genómica, Centro Universitario de Ciencias de la Salud, Universidad de Guadalajara, Guadalajara 44340, Jal, Mexico

**Keywords:** Alzheimer’s disease, early-onset, late-onset, eQTL, gene regulation, transcriptomics, precision medicine

## Abstract

**Background/Objectives:** Alzheimer’s disease (AD) exhibits marked genetic heterogeneity between early-onset (EOAD) and late-onset (LOAD) forms. EOAD is typically associated with highly penetrant variants, whereas LOAD follows a polygenic architecture dominated by non-coding variation. However, the tissue-specific regulatory consequences of these variants remain insufficiently characterized. This study aimed to compare the regulatory genomic architectures underlying EOAD and LOAD using a multi-tissue integrative approach. **Methods:** GWAS-associated variants for EOAD and LOAD were retrieved from the GWAS Catalog using a relaxed significance threshold (*p* < 1 × 10^−5^). Variants were functionally annotated and integrated with GTEx v8 eQTL data across 13 neurologically relevant tissues and peripheral blood. Regulatory effects were evaluated using eQTL slope estimates. Basal gene expression patterns were assessed using GTEx RNA-seq data, and protein–protein interaction and functional enrichment analyses were performed using the STRING database. **Results:** A total of 287 variants were analyzed (32 EOAD, 255 LOAD), with minimal overlap. EOAD exhibited a highly focal regulatory profile, identifying *GSE1* as the sole eQTL-regulated gene, restricted to the dorsolateral prefrontal cortex (BA9). In contrast, LOAD displayed a broad multi-tissue regulatory architecture involving *APH1B*, *APOE*, *CEP63*, and *HAVCR2*, with heterogeneous tissue-specific effects. LOAD-regulated genes converged on pathways related to γ-secretase activity, amyloid precursor protein processing, and Notch signaling, whereas *GSE1*-associated interactions were enriched for chromatin organization and epigenetic repression. **Conclusions:** EOAD and LOAD exhibit distinct regulatory genomic architectures, with EOAD characterized by focal, region-specific regulation and LOAD by widespread, tissue-dependent effects, highlighting stage-specific molecular mechanisms contributing to AD heterogeneity.

## 1. Introduction

Alzheimer’s disease (AD) is the leading cause of dementia, accounting for 60–80% of cases worldwide. In 2024, an estimated 6.9 million Americans aged 65 and older are affected, a number projected to rise to 13.8 million by 2060 without medical breakthroughs [1]. The global prevalence of dementia, including AD, was about 55 million in 2019 and is expected to triple by 2050, imposing significant socioeconomic burdens through caregiving demands and healthcare costs [2]. Despite advances in diagnostics, including biomarkers for amyloid and tau pathology, and recent FDA approvals of anti-amyloid monoclonal antibodies such as lecanemab and donanemab, current therapeutic options provide only modest slowing of cognitive decline in selected patients at early stages of Alzheimer’s disease, and no curative treatments are currently available [3,4,5].

Clinically, AD is classified as early-onset AD (EOAD), with symptoms before age 65, and late-onset AD (LOAD), with symptoms after age 65 [6]. EOAD often progresses rapidly and presents atypical features such as executive dysfunction, visuospatial impairments, or language deficits, while LOAD typically manifests gradual memory loss [7]. These differences extend to neuropathology and genetics, with EOAD showing more aggressive neuronal loss and LOAD following a slower course [8].

At the molecular level, AD is characterized by extracellular amyloid-β (Aβ) plaques and intraneuronal hyperphosphorylated tau tangles, which disrupt synaptic function and axonal transport, leading to neurodegeneration [3]. These hallmarks trigger microglial activation, astrogliosis, chronic inflammation, oxidative stress, and cytokine dysregulation, perpetuating a cycle of neuronal damage [9]. Vascular factors, metabolic disturbances, and immune responses further contribute to this multifactorial pathogenesis [3].

Genetically, EOAD is mainly caused by rare, high-penetrance autosomal dominant mutations in *APP*, *PSEN1*, and *PSEN2*, which increase Aβ production and aggregation, resulting in familial clustering and earlier onset [10]. In contrast, LOAD is polygenic, with *APOE* ε4 as the primary risk allele, along with common variants at loci such as *TREM2*, *CLU*, *BIN1*, *PICALM*, and *ABCA7*, which are involved in immunity, lipid homeostasis, endocytosis, and synaptic integrity [11]. Genome-wide association studies reveal partial overlap between EOAD and LOAD genetics, including age-of-onset modifiers [12].

Many AD risk variants are located in non-coding regions, influencing disease through gene expression regulation rather than protein changes, often in a tissue-specific manner [13]. Expression quantitative trait loci (eQTL) analyses link these variants to transcript levels, helping prioritize causal genes and pathways [14]. However, comparative eQTL studies across EOAD and LOAD in multiple tissues are limited, especially for brain regions and blood using resources such as GTEx [15].

In this study, we conducted an integrative multi-tissue regulatory genomics analysis to directly contrast EOAD and LOAD. By combining GWAS-derived variants with eQTL data from GTEx across neurologically relevant brain regions and peripheral blood, we aimed to: (i) characterize and compare the genomic distribution and functional annotation of EOAD- and LOAD-associated variants; (ii) identify tissue-specific and shared eQTL-regulated genes distinguishing early- and late-onset disease; and (iii) functionally contextualize prioritized genes using gene expression profiling and protein–protein interaction network analyses. Through this framework, we sought to define age-at-onset-specific regulatory architectures underlying Alzheimer’s disease and to prioritize molecular pathways relevant to future functional and translational studies [11].

## 2. Materials and Methods

To investigate the regulatory effects of disease-associated variants, GWAS-derived rsIDs were integrated with expression quantitative trait locus (eQTL) data from the Genotype-Tissue Expression (GTEx) project, version 8 (https://gtexportal.org/home/; accessed 30 October 2025). Lead cis-eQTLs were obtained from the v8.egenes.txt.gz files, which report the most significant regulatory variant per gene and tissue.

Variant matching was performed by aligning GWAS rsIDs with the GTEx identifier rs_id_dbSNP151_GRCh38p7. Only exact rsID matches were retained. For each variant–gene–tissue pair, the regulated gene (gene_name), nominal *p*-value (pval_nominal), multiple-testing-corrected value (qval), and effect size (slope) were extracted. Slope values represent the magnitude and direction of the regulatory effect on gene expression, with positive values indicating increased expression per copy of the alternate allele and negative values indicating decreased expression.

### 2.1. Tissue Selection

Analyses focused on tissues with established relevance to Alzheimer’s disease pathophysiology. Thirteen central nervous system and peripheral tissues were included: cerebral cortex, hippocampus, frontal cortex (BA9), anterior cingulate cortex (BA24), amygdala, caudate, cerebellum, cerebellar hemisphere, nucleus accumbens, putamen, hypothalamus, substantia nigra, and whole blood.

### 2.2. Data Consolidation and Expression Profiling

Significant GWAS–eQTL overlaps were compiled into two independent datasets corresponding to EOAD and LOAD. Basal gene expression levels, reported as transcripts per million (TPM), were obtained from GTEx RNA-seq data to assess tissue-specific expression profiles of prioritized genes.

### 2.3. Protein–Protein Interaction and Functional Enrichment Analysis

Protein–protein interaction (PPI) networks were constructed using the STRING database (version 11.5) and applying a minimum interaction confidence score of 0.4. Network topology was evaluated by calculating the number of observed interactions, expected interactions, enrichment *p*-values, average node degree, and local clustering coefficients.

Functional enrichment analyses were performed for Gene Ontology categories (biological process, molecular function, and cellular component), KEGG pathways, Reactome pathways, and disease associations. These analyses provided biological context for genes identified through the multi-tissue eQTL framework.

## 3. Results

### 3.1. Genomic Characterization of Variants Associated with EOAD and LOAD

To characterize the differential genetic architecture between EOAD and LOAD, a total of 287 GWAS-derived variants were jointly analyzed. Of these, 32 variants were associated with EOAD, while LOAD showed a considerably higher burden, with 255 associated variants.

The 32 EOAD-associated variants displayed a heterogeneous distribution across the autosomal chromosomes. Chromosomes 2 (*n* = 7) and 17 (*n* = 4) harbored the highest proportion of signals, while chromosomes 1, 6, 10, 11, 14, 18, and 19 each presented only a single variant.

The five most significant variants associated with early-onset Alzheimer’s disease were distributed across four autosomal chromosomes and exhibited a genomic pattern concentrated in specific regions. The most prominent signal was variant rs769449 (*p* = 3.0 × 10^−15^), located on chromosome 19 and linked to the *APOE* gene, widely recognized as a key locus in the pathophysiology of the disease. This was followed by variant rs55889290 (*p* = 4.0 × 10^−7^) on chromosome 16, annotated in the *CES5A* gene. On chromosome 17, variant rs62061022 (*p* = 6.0 × 10^−7^) was identified and associated with *PIMREG,* while variant rs33928449 (*p* = 1.0 × 10^−6^), also on this chromosome, was related to *EXOC7*. Finally, variant rs11903348 (*p* = 7.0 × 10^−7^) was found on chromosome 2, in a region spanning genes such as *RPL38P2* and *LIN*. Collectively, these signals represent the most robust associations within the EOAD set and reflect a more focal genetic architecture, characterized by specific loci with significantly enriched effects (Table 1).

The 255 variants associated with LOAD showed a broader and more homogeneous distribution across the genome, with marked enrichment on chromosomes 2 (*n* = 22), 19 (*n* = 21), and 7 (*n* = 19). Chromosome 21 had the fewest variants, with only two reported signals. Neither EOAD nor LOAD exhibited variants on the sex chromosomes.

As with EOAD, five principal variants associated with LOAD were identified. There was a marked predominance on chromosome 19, reflecting the strong contribution of the *APOE* locus to the genetic architecture of this condition. The most robust association was rs429358 (*p* = 1.00 × 10^−300^), located in the *APOE* gene, followed by rs7412 (*p* = 3.00 × 10^−105^), also in the same gene and representing the second most prominent signal. Additionally, variant rs4663105 (*p* = 4.00 × 10^−58^), located on chromosome 2 and related to the genes *NIFKP9* and *BIN1*, provided a significant signal outside the *APOE* axis. Another relevant variant was rs10119 (*p* = 2.00 × 10^−57^), located on chromosome 19 within the *TOMM40* gene, a locus closely linked to the *APOE* cluster. Finally, variant rs1582763 (*p* ≈ 6.03 × 10^−7^), located on chromosome 11 and annotated in *MS4A4A*, was identified, expanding the genetic diversity observed among the principal associations (Table 1).

**Figure 1:**
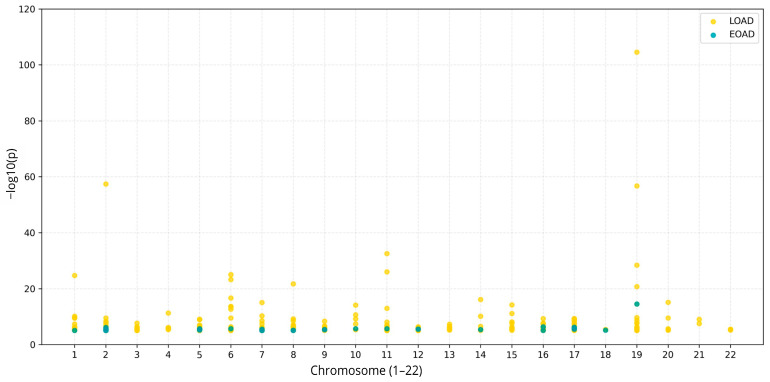
Genomic distribution of variants associated with early-onset Alzheimer’s disease (EOAD, blue) and late-onset Alzheimer’s disease (LOAD, yellow) across the 22 autosomal chromosomes. Values represent the strength of association, expressed as −log_10_(p). The figure shows the relative genomic burden and chromosomal distribution of risk variants in each disease group. Figure created using Python version 3.10.

Figure 1 shows the genomic distribution of variants associated with EOAD and LOAD across the 22 autosomal chromosomes.

Table 1 summarizes the total number of variants per chromosome and highlights the most statistically significant variant(s) for each chromosome, including rsID, annotated gene(s), and association *p*-values. For clarity, only the top ten variants per disease group are shown.

**Figure 2:**
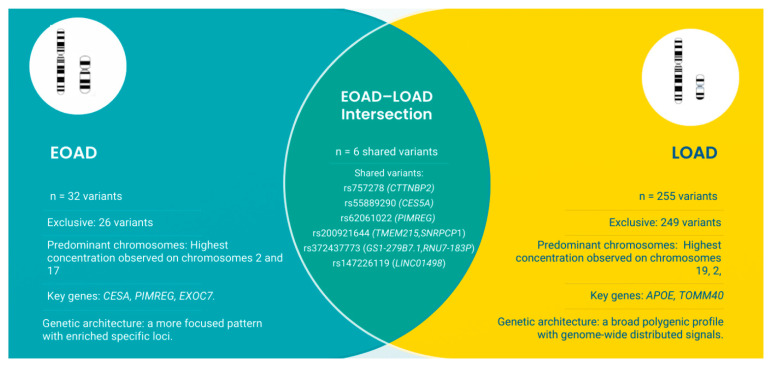
Venn diagram summarizing the differences and overlap between early-onset Alzheimer’s disease (EOAD) and late-onset Alzheimer’s disease (LOAD). EOAD included 32 associated variants (26 exclusive), while LOAD included 255 variants (249 exclusive). Six variants were shared between the two groups, reflecting loci with potential cross-phenotypic relevance. Figure created using Canva pro version 2.5.

The direct comparison between the two sets allowed quantification of the degree of genetic overlap: 26 variants were exclusive to EOAD, 249 were exclusive to LOAD, and 6 variants were shared between the conditions, as shown in Figure 2.

**Figure 3:**
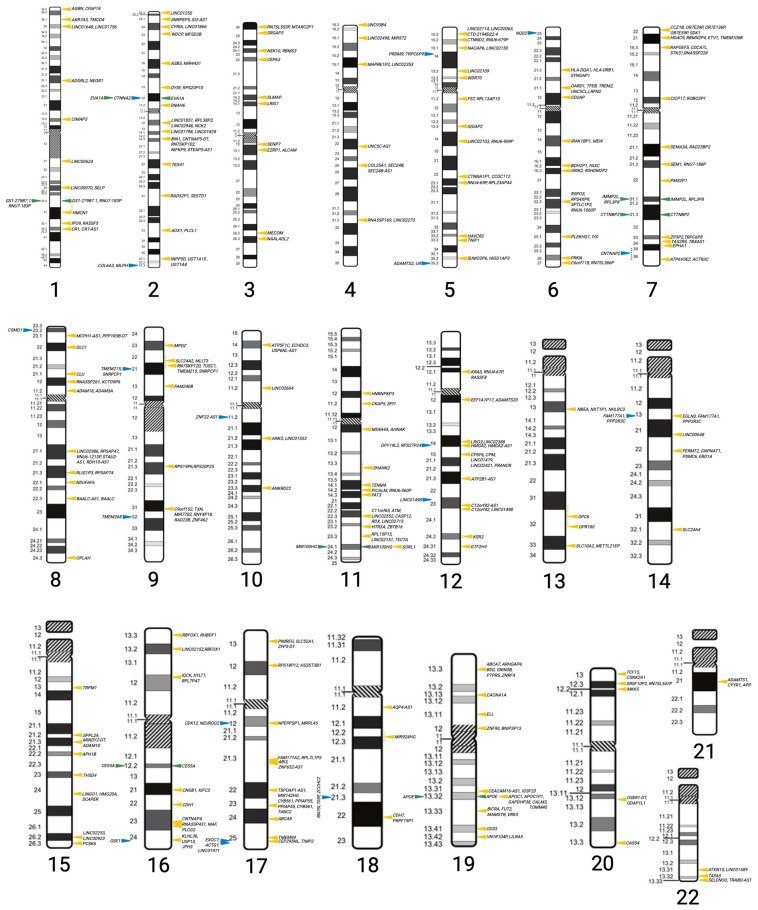
Chromosomal distribution of genes associated with Alzheimer’s disease identified through genome-wide association studies (GWAS). The figure shows the genomic locations of genes associated with late-onset Alzheimer’s disease (LOAD, yellow), early-onset Alzheimer’s disease (EOAD, blue), and genes shared by both forms of the disease (green). Visualization adapted from the Atlas of Genetics and Cytogenetics in Oncology and Hematology and created with BioRender. Gyc, L. (2026) https://BioRender.com/qqetrph (accessed on 30 October 2025).

To visualize the genomic distribution of genes identified through GWAS, their locations were mapped across the 22 autosomal chromosomes (Figure 3). This representation allows observation of the overall genetic architecture of Alzheimer’s disease-associated loci and facilitates identification of regions with a higher concentration of risk-associated genes.

### 3.2. Functional Annotation and Gene Distribution of GWAS Variants

Classification of the identified variants allowed differentiation of their genomic locations and molecular effects. In both EOAD and LOAD, most variants were clustered in intergenic and intronic regions, while other categories where less represented. The complete distribution of annotated consequences for each group is presented in the following table (Table 2):

Distribution of genome-wide association studies (GWAS-derived) variants according to their most severe predicted functional consequence in early-onset (EOAD) and late-onset Alzheimer’s disease (LOAD). Functional categories were assigned based on variant annotation, and counts represent the number of variants in each consequence class for each disease group.

Certain genes appear more frequently than others, indicating a higher concentration of associated records or events in these genomic regions. In the EOAD analysis, duplication was limited to a few elements: *CSMD1* was recorded twice in the same manner, while the *ADAMTS2–U4* pair was detected twice with the order reversed, representing the same gene combination in both cases.

In contrast, the accumulation of entries was more pronounced in LOAD. The *NBEA* gene showed the highest recurrence, appearing three times, followed by a group of genes (*ABCA7*, *APOE*, *CD33*, *SCAPER*, and *TREM2*) that were each identified in two independent records. Additionally, the gene pairs *RPS19P6–RPS20P25* and *CYB561–PPIAP55* each appeared twice.

### 3.3. Identification of eQTL-Regulated Genes in Tissues Associated with EOAD and LOAD

Genes with evidence of regulation mediated by eQTL variants were identified across multiple brain tissues and in peripheral blood. In late-onset disease, *CEP63* showed the broadest regulatory pattern, with signals detected in the Brain Anterior Cingulate Cortex, Brain Caudate, Brain Cerebellum, Brain Cerebellar Hemisphere, and Brain Hypothalamus. In the Brain Amygdala, regulation associated with *PMS2P1* was observed, while regulatory effects on *CR1-AS1/CR1* were identified in the Brain Cortex.

In the Brain Frontal Cortex BA9, eQTL variants were associated with *APH1B*, *APOE*, and *KLHL36/USP10*. In the Brain Putamen, regulatory signals for *APH1B*, *APOE*, and *HAVCR2* were detected, while in the Brain Nucleus Accumbens, regulation of *ABCA7* was identified. Additionally, eQTLs for *HAVCR2* were observed in the Brain Hypothalamus, and regulatory signals for *APH1B* and *HAVCR2* were found in Whole Blood.

Each gene presented a single regulatory variant per tissue, except for *APOE*, *APH1B*, *HAVCR2*, and *CEP63*, which appeared in multiple regions due to independent signals from different anatomical areas.

In contrast, in early-onset disease, only one gene with evidence of eQTL regulation was identified: *GSE1*, associated with variant rs58675609, detected exclusively in the frontal cortex (BA9). No additional eQTL signals were observed in other tissues, nor were there overlaps with those identified in LOAD. This pattern highlights a clear difference in the number and tissue distribution of regulatory signals between EOAD and LOAD. The complete relationship between genes, tissues, and variants is detailed in Table 3.

This contrast between disease stages indicates that, while the late stage is characterized by a broad regulatory architecture distributed across multiple brain regions, the early stage exhibits a highly focal signal that may represent a primary or initiating event in the pathobiological cascade. The complete set of gene–tissue–variant relationships is summarized in Table 3.

For each gene, the associated regulatory variant (rsID), the tissue in which regulation was detected using GTEx v8 data, and the corresponding disease group are indicated. EOAD: early-onset Alzheimer disease; LOAD: late-onset Alzheimer disease. SNP: single nucleotide polymorphism.

To visualize the magnitude and direction of the identified regulatory effects, we analyzed the slope values of each eQTL variant across the different tissues available in GTEx. Because the slope represents the expected change in gene expression per alternate allele, its distribution indicates whether the variants tend to increase or decrease the expression of the associated genes.

**Figure 4:**
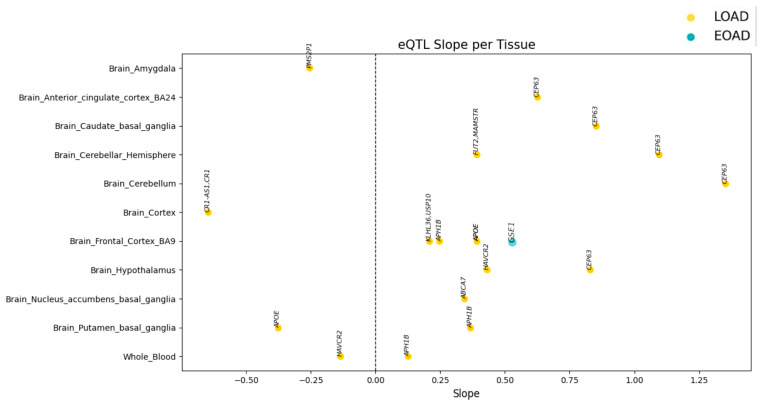
Distribution of regulatory effects (slope values) of eQTL variants associated with genes implicated in Alzheimer’s disease, shown by tissue. Each point represents a variant–gene pair identified in GTEx, positioned according to the estimated slope value reflecting its effect on gene expression. The vertical dashed line indicates slope = 0, distinguishing positive effects (increased expression) from negative effects (decreased expression). Figure created using Python version 3.10.

Figure 4 shows these effects by tissue, enabling comparison of regulatory patterns across brain regions and peripheral blood, and allowing identification of genes with particularly high, consistent, or divergent effects among tissues.

The regulatory effects of eQTL variants were evaluated in genes detected in more than one tissue (*APH1B*, *APOE*, *CEP63*, and *HAVCR2*) to determine directional consistency and variations in effect magnitude across brain regions and peripheral blood. Overall, the data revealed heterogeneous regulatory patterns depending on the gene analyzed.

For *APH1B*, slope values were consistently positive across the three tissues in which regulation was detected (Brain Frontal Cortex BA9: 0.247; Brain Putamen: 0.366; Whole Blood: 0.125). This indicates a homogeneous regulatory effect in direction, with moderate variation in effect strength, the strongest effect observed in the putamen and the weakest in peripheral blood.

In contrast, *APOE* exhibited a mixed pattern: in Brain Frontal Cortex BA9 the effects were clearly positive (0.392), whereas in Brain Putamen, the associated variant showed a negative effect (−0.377). This finding indicates an inversion of the regulatory effect depending on the tissue, suggesting that *APOE* regulation is highly tissue-context dependent and that the same variant may activate or repress gene expression depending on the specific cellular environment.

The *CEP63* gene showed the greatest overall magnitude of regulatory effect and the most consistent values across tissues, all in the positive direction. Slopes of 0.623 were observed in the anterior cingulate cortex, 0.851 in the caudate, 1.094 in the cerebellar hemisphere, 1.351 in the cerebellum—the highest value across the entire dataset—and 0.828 in the hypothalamus. This pattern indicates robust and sustained gene activation across multiple brain regions, with a clear intensification of the effect in cerebellar structures.

Finally, *HAVCR2* showed a divergent pattern similar to *APOE*: it had a strongly positive effect in the hypothalamus (0.430), but a negative effect in peripheral blood (−0.136). This inversion further confirms that *HAVCR2* regulation also depends on tissue type.

This analysis enables prioritization of genes with consistent regulation (indicating a higher likelihood of cross-tissue relevance) and genes with divergent regulation (potential modulators specific to vulnerable regions in Alzheimer’s disease).

### 3.4. Basal Expression Profiles of eQTL-Regulated Genes in EOAD and LOAD

**Figure 5:**
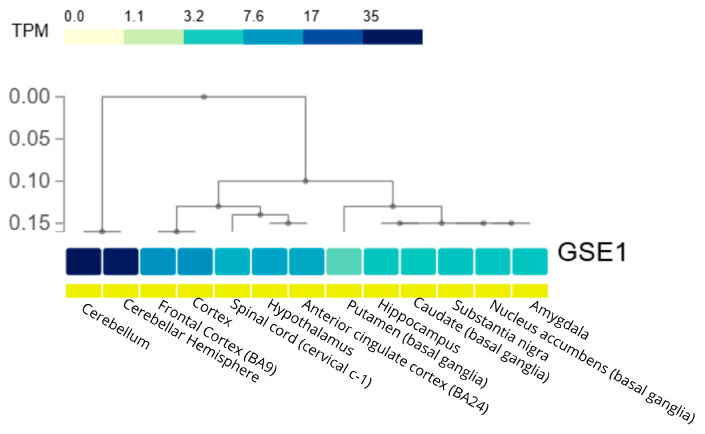
Brain expression of *GSE1*, an eQTL-regulated gene in early-onset Alzheimer’s disease. Hierarchical heatmap showing *GSE1* expression levels (transcripts per million, TPM) across human brain regions using data from the GTEx project. Brain regions are grouped by hierarchical clustering based on expression similarity. The color scale represents increasing TPM values (from light to dark blue), highlighting regional variation in *GSE1* expression. Figure created using GTEx data v10.

Since the EOAD-specific eQTL analysis identified *GSE1* as the only gene regulated at this stage, its basal expression pattern across different human brain regions was evaluated (Figure 5). For this purpose, gene expression data (TPM) from the GTEx project were used, and a hierarchical heatmap was constructed to characterize the regional distribution of *GSE1* in the brain.

**Figure 6:**
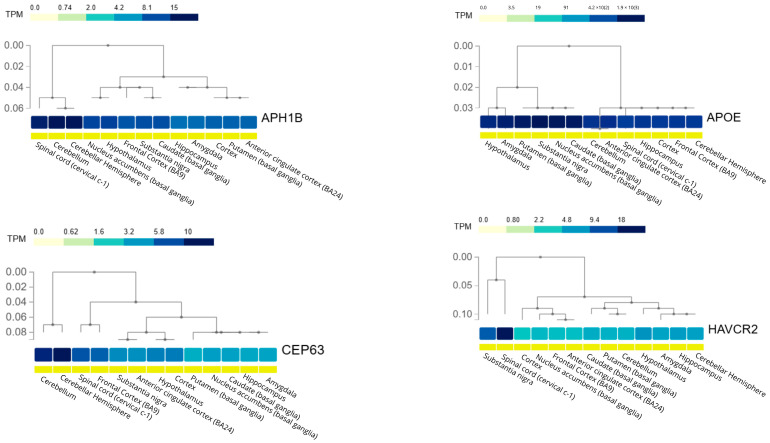
Gene expression profiles of *APH1B*, *APOE*, *CEP63*, and *HAVCR2* across human brain regions. Hierarchical heatmaps display gene expression levels (transcripts per million, TPM) across brain regions using data from the GTEx project. Each panel represents to a single gene, with tissues grouped by hierarchical clustering based on expression similarity. The color scale indicates increasing TPM values (from light to dark blue), and dendrograms illustrate transcriptional relationships among brain regions, highlighting tissue-specific expression patterns. Figure created using GTEx data.

The basal expression patterns of genes identified as eQTL-regulated in the context of LOAD were also evaluated (Figure 6). Hierarchical heatmaps were generated for *APH1B*, *APOE*, *CEP63*, and *HAVCR2* across multiple human brain regions. This analysis enabled comparison of the relative abundance of these genes among tissues and exploration of the transcriptional organization of different brain regions, providing biological support for tissue-specific interpretation of the regulatory effects observed in LOAD.

### 3.5. Protein–Protein Interaction Networks of eQTL-Regulated Genes in EOAD and LOAD

**Figure 7:**
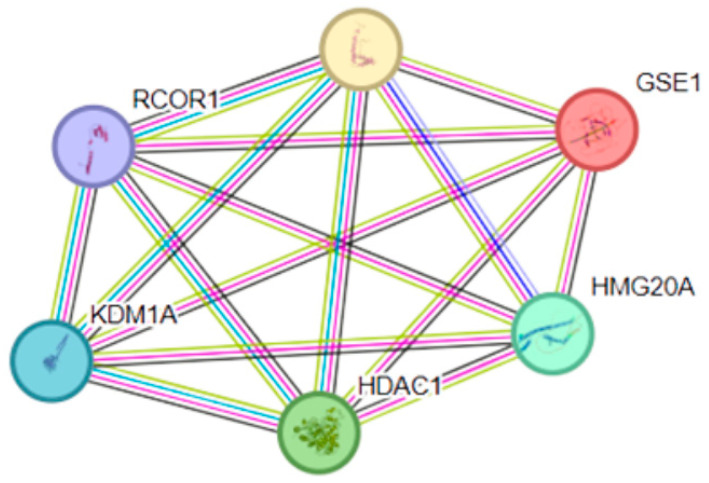
Protein–protein interaction (PPI) network centered on GSE1. The figure shows the interaction network of the GSE1 protein and its associated proteins: RCOR1, KDM1A (LSD1), HDAC1, HMG20A, and HMG20B. All nodes are shown as colored and filled, indicating that they correspond to the query proteins and first-shell interactors with known or predicted 3D structures. The protein–protein interaction network was generated using the STRING database. Edges represent different types of interaction evidence, with colors denoting their source: curated databases (black), experimentally determined interactions (pink), gene neighborhood (green), gene fusions (red), gene co-occurrence (blue), text mining (yellow), co-expression (light blue), and protein homology (purple). The dense connectivity and overlap of multiple evidence types indicate strong functional and regulatory coupling among these proteins, particularly within chromatin remodeling and transcriptional regulation complexes.

As *GSE1* was identified as the eQTL-regulated gene in EOAD, its functional context was assessed through protein–protein interaction analysis. GSE1 was part of a highly interconnected network with HMG20A, HMG20B, RCOR1, KDM1A, and HDAC1, forming a module of six nodes and 15 observed interactions, significantly more than the six interactions expected by chance (PPI enrichment *p* = 0.00049). The local clustering coefficient was 1, indicating a highly cohesive organization. (Figure 7)

Functional enrichment analysis of this network revealed a significant overrepresentation of biological processes related to chromatin organization (GO:0006325), histone H4 deacetylation (GO:0070933), and negative regulation of protein sumoylation. Additionally, enrichment of key epigenetic complexes was identified, including the CoREST complex, the Sin3 complex, and histone deacetylase complexes, which actively participate in chromatin-dependent transcriptional repression.

**Figure 8:**
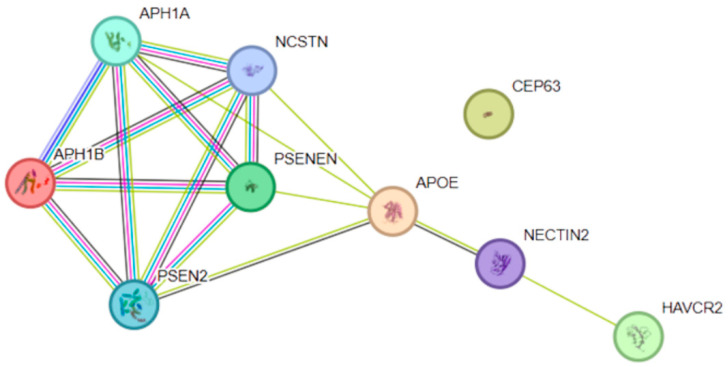
Protein–protein interaction (PPI) network of prioritized genes associated with late-onset Alzheimer’s disease. The network shows functional interactions among APH1A, APH1B, PSEN1, PSEN2, NCSTN, APOE, NECTIN2, HAVCR2, and CEP63, constructed using the STRING database. Edges represent different types of evidence supporting protein–protein interactions, with edge colors indicating the source of evidence: curated databases (black), experimentally determined interactions (pink), gene neighborhood (green), gene fusions (red), gene co-occurrence (blue), text mining (yellow), co-expression (light blue), and protein homology (purple). The presence of multiple overlapping edge types indicates strong and well-supported interactions, particularly within pathways related to proteolytic processing, membrane protein regulation and neurodegenerative disease mechanisms.

The protein–protein interaction network analyzed for eQTL-regulated genes in LOAD consisted of 9 nodes and 16 interactions (Figure 8), with an average node degree of 3.56 and a mean local clustering coefficient of 0.689, indicating high interconnectivity among the included proteins. The number of observed interactions was significantly higher than expected by chance (16 observed vs. 6 expected), supported by a highly significant PPI enrichment value (*p* = 0.000228).

Functional enrichment analysis revealed strong overrepresentation of biological processes related to amyloid precursor protein metabolism and amyloid-β peptide generation. The most significantly enriched terms included “amyloid precursor protein metabolic process” (6 of 23 genes; FDR = 7.67 × 10^−12^), “amyloid-beta formation” (5 of 9 genes; FDR = 7.98 × 10^−11^), and “Notch receptor processing” (5 of 9 genes; FDR = 7.98 × 10^−11^). Enrichment was also found for processes related to membrane protein proteolysis and Notch signaling.

Within the molecular function category, significant enrichment was observed for endopeptidase activator activity and aspartic endopeptidase activity with intramembrane cleavage, consistent with the function of the γ-secretase complex. At the cellular component level, the term “gamma-secretase complex” showed one of the strongest signals (5 of 7 genes; FDR = 8.24 × 10^−12^), along with compartments associated with membranes, endosomes, and the Golgi apparatus.

Pathway analysis identified significant enrichment in the Notch signaling pathway (KEGG hsa04330) and the Alzheimer’s disease pathway (KEGG hsa05010), as well as multiple Reactome pathways related to non-canonical NOTCH3 activation, NOTCH4 signaling, and regulated proteolysis. WikiPathways terms also highlighted Notch signaling and Alzheimer’s disease-related processes.

Disease association analyses showed significant enrichment for Alzheimer’s disease, amyloid plaques, neurofibrillary tangles, and related human phenotypes.

## 4. Discussion

### 4.1. Regulatory Genetic Architecture Differences Between EOAD and LOAD

The analyses indicate that LOAD is characterized by a broad regulatory architecture involving significantly more associated variants than EOAD. This contrast aligns with previous genetic evidence showing that EOAD is primarily driven by a limited set of highly penetrant variants, while LOAD reflects a complex polygenic configuration composed of numerous loci with modest individual effects [16,17,18,19].

Differences between EOAD and LOAD were also evident at the chromosomal level. LOAD-associated variants were distributed across many chromosomes, while EOAD signals were confined to a more restricted subset. Importantly, chromosomal contribution should not be interpreted solely based on variant counts. In EOAD, chromosomes with few detectable associations may still contain localized regulatory regions where limited genetic variation exerts disproportionate influence on critical regulatory elements, rather than indicating reduced biological relevance.

In LOAD, chromosomes 2 and 19 emerged as major contributors, largely due to the influence of loci such as *BIN1* and the *APOE/TOMM40* region. These loci are implicated in processes central to Alzheimer’s disease pathophysiology, including endocytic trafficking, lipid homeostasis, immune regulation, and mitochondrial function [16,17,18,19,20]. Additionally, chromosome 7 has repeatedly been identified in genome-wide association and linkage analyses, supporting the presence of loci that may act as genetic modifiers rather than primary disease determinants [16,21].

At the gene level, EOAD was defined by a relatively small set of associated genes, whereas LOAD displayed a substantially broader gene-level signal, with only six genes shared between the two conditions. Rather than implying limited overlap of biological pathways, this pattern suggests convergence on a restricted set of core genes alongside divergence across a broader landscape of context-dependent or modifier loci. This configuration is consistent with conceptual models proposing Alzheimer’s disease as a heterogeneous syndrome characterized by variable genetic penetrance rather than a single, uniform genetic entity [19,22,23].

No significant regulatory signals were detected on the sex chromosomes in either EOAD or LOAD. Although this finding differs from studies reporting sex-specific genetic contributions to Alzheimer’s disease risk [24], the absence of detectable signals does not exclude biological relevance. Instead, it may reflect regulatory mechanisms not captured by conventional eQTL frameworks, such as sex-dependent effects, X-chromosome inactivation, hormonal modulation, or epigenetic regulation [25,26].

### 4.2. Functional Annotation Highlights Non-Coding and Recurrent Gene-Level Signals in EOAD and LOAD

Functional annotation showed that most GWAS-associated variants in both EOAD and LOAD are located in intronic or intergenic regions, supporting a model in which genetic risk is largely conveyed through regulatory processes rather than alterations of protein-coding sequences. This distribution aligns with genome-wide evidence highlighting the contribution of non-coding variation to the genetic architecture of complex neurodegenerative disorders [27,28].

At the gene level, EOAD showed limited recurrence of associated genes, consistent with a more constrained regulatory configuration. In contrast, LOAD exhibited a greater accumulation of gene-level signals, with multiple genes identified across independent annotations. This recurrence is unlikely to reflect technical redundancy and instead suggests genomic regions experiencing increased regulatory load or convergent genetic effects. Notably, several of these recurrent genes have previously been linked to Alzheimer’s disease susceptibility, reinforcing their interpretation as modifier or risk loci within the polygenic landscape of LOAD [29].

### 4.3. Broad and Mult-Systemic eQTL Architecture in LOAD

APH1B and HAVCR2 were among the few genes exhibiting regulatory effects in both brain tissues and whole blood, and both loci have been implicated in large-scale genome-wide association studies of Alzheimer’s disease [30]. Independent integrative studies have reported associations between dysregulated *APH1B* expression in peripheral blood and neuroimaging markers, including brain atrophy and amyloid-β deposition, indicating that its regulatory effects extend beyond the central nervous system [31,32]. While peripheral gene expression cannot be assumed to directly reflect central nervous system processes [33], the presence of genetically driven regulation across distinct compartments suggests involvement in systemic mechanisms contributing to Alzheimer’s disease pathology. This observation underscores the potential utility of peripheral tissues for capturing genetically mediated regulatory effects when brain tissue access is limited.

Further evidence supporting the distributed regulatory architecture of LOAD comes from the identification of established Alzheimer’s disease loci with strong signals of genetic regulation and co-localization across immune and glial cell types. Variants at loci including *PICALM*, *CR1*, *SLC24A4/RIN3*, and *USP6NL* showed robust summary-based Mendelian randomization (SMR) associations with high posterior probabilities of shared causal variants (PP.H4.abf ≈ 0.99), consistent with previous large-scale integrative analyses [34].

These regulatory signals were detected predominantly in microglial, oligodendroglial, and immune-related cellular contexts, reinforcing a model in which LOAD risk is mediated through widespread, cell type-specific regulatory effects rather than localized perturbations. This pattern contrasts with the regulatory landscape observed for EOAD in the present study, where genetically regulated expression was confined to a single gene (*GSE1*) and a single cortical region, highlighting fundamental differences in the regulatory organization underlying early- and late-onset forms of Alzheimer’s disease.

### 4.4. Highly Focal eQTL Regulation in EOAD Suggests Early Regulatory Vulnerability

In contrast to the broadly distributed regulatory architecture observed in LOAD, EOAD displayed a sharply delimited eQTL profile. Genetically regulated expression was confined to *GSE1* and restricted to the dorsolateral prefrontal cortex (BA9), underscoring a clear divergence between the regulatory landscapes of early- and late-onset disease. The absence of regulatory signals across additional brain regions or peripheral tissues supports a model in which EOAD is characterized by localized regulatory perturbations rather than pervasive transcriptional reorganization.

This pattern is consistent with integrative frameworks proposing that early molecular alterations in Alzheimer’s disease emerge in a region-specific manner, reflecting selective vulnerability of discrete neural networks instead of global dysregulation [33]. The restriction of genetically regulated expression to BA9 is particularly notable given the involvement of this region in executive function, behavioral regulation, and emotional processing—domains frequently affected during the early neuropsychiatric stages of Alzheimer’s disease [35,36].

Although GSE1 has not been extensively studied in the context of Alzheimer’s disease, independent integrative genomic approaches that combine regulatory prioritization and molecular quantitative trait locus analyses have identified GSE1 as a candidate gene within AD risk frameworks [37]. Its emergence in the EOAD regulatory landscape supports the idea that early-onset disease may be shaped by narrowly defined, context-dependent regulatory events.

Importantly, the limited number of detectable eQTL signals in EOAD should not be interpreted as diminished biological relevance. Instead, this pattern aligns with models suggesting that EOAD and LOAD follow partially divergent biological and temporal trajectories, with early-onset forms driven by upstream regulatory mechanisms rather than representing an accelerated manifestation of late-onset disease [29,35]. In addition, EOAD exhibits substantial heterogeneity in genetic penetrance, and prior studies have identified genetic modifiers capable of delaying onset or attenuating clinical expression even among carriers of pathogenic variants [22,38]. Such modifying effects may further contribute to the scarcity of steady-state eQTL signals detected in EOAD by influencing regulatory processes in a cell-type- and context-specific manner that is not fully captured by bulk transcriptomic datasets.

### 4.5. GSE1 and Chromatin-Associated Complexes as Early Regulatory Nodes in EOAD

Functional analyses provide insight into molecular pathways through which GSE1-associated regulation may influence early vulnerability in EOAD. Protein–protein interaction and functional enrichment analyses place GSE1 within chromatin-associated repression complexes involving CoREST, histone deacetylases (HDACs), and the histone demethylase KDM1A [39]. These complexes mediate transcriptional repression through coordinated chromatin remodeling and histone modification, representing key regulatory nodes in gene expression control.

CoREST-, HDAC-, and KDM1A-containing complexes play essential roles in maintaining neuronal identity and regulating activity-dependent transcriptional programs. Experimental disruption of HDAC- and KDM1A-related pathways increases neuronal susceptibility by altering transcriptional programs required for synaptic stability, stress responses, and neuronal survival [40,41]. In this context, GSE1-associated regulation may reflect an early imbalance in chromatin-mediated control mechanisms that precedes broader neurodegenerative changes.

This interpretation aligns with models proposing that EOAD involves early disturbances in regulatory systems that operate upstream of widespread transcriptional alterations. Such perturbations are likely highly context-dependent and restricted to specific cell populations, which may explain their limited detectability in conventional multi-tissue eQTL analyses.

Instead of providing experimental functional validation of EOAD mechanisms, this study situates its findings within the framework of established biological pathways. *GSE1* is a recognized interactor in chromatin-associated co-repressor complexes, particularly those containing HDAC1 and CoREST, which integrate deacetylase, demethylase, and other regulatory activities essential for transcriptional regulation and maintenance of chromatin states [39,42]. These complexes have well-documented roles in the regulating gene expression programs involved in neuronal differentiation and maintenance of neuronal identity [43]. Dysregulation of such chromatin-modifying systems has been associated with altered chromatin dynamics and increased neuronal vulnerability in neurodegenerative contexts, including Alzheimer’s disease [44].

Consistent with this view, epigenetic and chromatin-remodeling mechanisms—such as histone modifications and changes in chromatin accessibility—have been repeatedly implicated in the pathophysiology of Alzheimer’s disease and age-related cognitive decline [41,44]. From this perspective, identifying GSE1 as a focal regulatory signal highlights a potential early regulatory node within known epigenetic control systems, rather than providing direct functional evidence of EOAD-specific molecular mechanisms. Previous work has shown that epigenetic mechanisms can contribute to disease susceptibility in the absence of extensive steady-state gene expression changes, particularly in neurodevelopmental and neurodegenerative disorders [33,44]. Together, these observations underscore the relevance of chromatin-associated regulatory mechanisms for understanding early pathogenic processes in EOAD.

### 4.6. Tissue-Dependent Regulatory Effects and Directional Heterogeneity of eQTLs in LOAD

The opposite regulatory directions observed for genes such as *APOE* and *HAVCR2* across tissues highlight the critical role of tissue context in interpreting eQTL effects [45]. These findings indicate that a single genetic variant can exert distinct, and sometimes opposing, regulatory influences depending on the cellular environment, a phenomenon extensively documented for *APOE* across brain regions and cell types [18,19]. Tissue-dependent regulation of this nature likely contributes to discrepancies reported across transcriptomic studies and cautions against oversimplified interpretations of gene expression changes in Alzheimer’s disease [46,47].

By contrast, genes such as *APH1B* showed a consistent direction of regulatory effect across brain regions and peripheral blood, indicating more stable cross-tissue regulatory influences. This pattern distinguishes *APH1B* from the context-dependent behavior observed for *APOE* and *HAVCR2*, illustrating that LOAD involves both coherent regulatory signals and highly tissue-specific effects.

*APOE* exemplifies this regulatory complexity. Despite its well-established role in Alzheimer’s disease susceptibility, its expression and genetically driven regulation vary substantially across brain regions and cell types and can exhibit opposing effects depending on the tissue examined [48,49]. This pronounced context dependence highlights the limitations of extrapolating regulatory inferences from single-tissue analyses and reinforces the value of multi-tissue frameworks for interpreting genetically mediated regulation in Alzheimer’s disease.

### 4.7. Functional Integration: Convergence of LOAD eQTL-Regulated Genes on γ-Secretase and Notch Signaling

Functional integration of eQTL-regulated genes associated with LOAD demonstrated convergence on canonical Alzheimer’s disease pathways, particularly those involving γ-secretase activity, amyloid precursor protein (APP) processing, and Notch signaling. These systems are functionally intertwined, as γ-secretase catalyzes the proteolytic cleavage of both APP and Notch receptors, linking amyloid-related processes with broader signaling pathways essential for neuronal integrity and maintenance [50,51,52].

Rather than implicating previously unrecognized biological pathways, this pattern indicates that genetic susceptibility in LOAD primarily modulates established Alzheimer’s disease mechanisms through regulatory variation distributed across multiple genes and tissues. This interpretation aligns with large-scale genetic studies showing that LOAD risk results from the cumulative effects of numerous variants converging on shared molecular processes, rather than from a small number of dominant drivers [16,19].

Collectively, these observations support a model in which LOAD is characterized by progressive dysregulation of core molecular pathways—including γ-secretase-mediated APP metabolism and Notch signaling—driven by context-dependent regulatory variation rather than uniform changes in gene expression.

### 4.8. Clinical and Neuropsychiatric Implications of Tissue-Specific Regulatory Effects

The detection of tissue-specific regulatory effects across multiple brain regions highlights the potential clinical relevance of genetically mediated regulatory variation in Alzheimer’s disease. Although the present data do not support direct inferences of causality between gene regulation and clinical manifestations, the spatial distribution of regulatory signals provides a biologically coherent framework for contextualizing disease-associated neuropsychiatric features.

Several regions showing regulatory effects in LOAD—including frontal cortical areas, limbic structures, basal ganglia, and the hypothalamus—are centrally involved in executive function, emotional processing, motivation, and behavioral regulation [53,54]. Altered function within these circuits has been consistently linked to neuropsychiatric manifestations such as apathy, depression, agitation, and impaired decision-making, which frequently accompany cognitive decline in Alzheimer’s disease [55,56].

The convergence of genetically regulated expression in regions involved in emotional and motivational processing suggests that regulatory variation may contribute to the selective vulnerability of neural circuits underlying neuropsychiatric symptoms. Rather than reflecting isolated molecular effects, these patterns align with network-based models of Alzheimer’s disease, in which distributed but context-dependent perturbations across interconnected regions shape behavioral and emotional phenotypes throughout disease progression [57,58].

Importantly, these observations do not imply direct genotype–phenotype relationships but highlight the interpretive value of tissue-resolved regulatory analyses. By mapping regulatory signals onto brain regions with well-defined functional domains, this approach provides a conceptual link between molecular genetic variation and the complex neuropsychiatric presentation of Alzheimer’s disease, supporting the utility of multi-tissue regulatory frameworks for understanding disease heterogeneity [45,47].

### 4.9. Translational Implications: Epigenetic Regulation as a Therapeutic Opportunity in EOAD

The findings of this study provide human genetic evidence supporting the prioritization of regulatory mechanisms with potential translational relevance, particularly in the context of EOAD. In contrast to the broadly distributed and multi-systemic regulatory architecture observed in LOAD, EOAD exhibited a sharply delimited eQTL profile restricted to a single gene and a specific cortical region. This divergence supports the interpretation that early pathogenic processes in EOAD are driven by regulatory mechanisms that are biologically distinct from those underlying late-onset disease [29,35].

The identification of *GSE1* as the only gene showing eQTL regulation in EOAD, with effects restricted to the dorsolateral prefrontal cortex (BA9), indicates an early and region-specific regulatory vulnerability. Given the established role of *GSE1* in CoREST-associated epigenetic repression complexes, genetically regulated variation in its expression may influence chromatin organization and transcriptional repression programs within a cortical region essential for executive control and emotional regulation [42,43].

Protein–protein interaction analyses further place GSE1 within a tightly interconnected module comprising KDM1A (LSD1), HDAC1, RCOR1, and HMG20A/B, core components of CoREST-related chromatin-modifying complexes. These assemblies play critical roles in histone modification, chromatin structure, and transcriptional repression, and are required for maintaining neuronal identity and regulating activity-dependent gene expression [42,43]. Disruption of epigenetic regulators within these complexes has been associated with increased neuronal susceptibility and neurodegenerative processes, including Alzheimer’s disease [33,44].

From a translational perspective, the alignment between EOAD-associated genetic regulation and epigenetic mechanisms with known pharmacological tractability suggests that epigenetic dysregulation may occur at early stages of EOAD pathogenesis. Unlike therapeutic strategies targeting downstream structural damage or protein aggregation, approaches based on genetically supported regulatory pathways provide a rational framework for intervention during earlier windows of disease susceptibility [59].

In contrast, LOAD is characterized by the convergence of multiple co-localized regulatory variants across immune- and glial-related pathways, indicating a fundamentally different regulatory organization. This contrast suggests that therapeutic strategies effective for EOAD are unlikely to be directly transferable to LOAD, highlighting the need for disease-stage-specific and age-at-onset-specific intervention paradigms.

Collectively, these results establish a genetic and bioinformatic framework that supports prioritizing epigenetic regulatory mechanisms as therapeutic opportunities in EOAD. By grounding intervention strategies in human genetic evidence, this study contributes to identifying biologically relevant targets for future functional and pharmacological investigations aimed at mitigating early neuronal vulnerability in early-onset Alzheimer’s disease.

## 5. Limitations

This study has some limitations that should be considered when interpreting the findings. First, the integrative analyses relied on association signals that did not consistently meet genome-wide significance thresholds. Although this approach is increasingly used in exploratory integrative genomics to identify potentially relevant regulatory mechanisms, it may increase susceptibility to false-positive associations. Therefore, the reported loci should be considered candidates requiring further validation.

Second, the analyses were based primarily on bulk tissue eQTL data from GTEx. While this resource provides broad coverage across tissues, bulk transcriptomic profiles may obscure cell-type-specific regulatory effects that are likely relevant in neurodegenerative diseases. Differences in sample size across tissues may also affect detection power, limiting direct comparisons between brain and peripheral tissues.

Third, the study does not include stratified analyses based on age at onset or datasets specifically enriched for early-onset Alzheimer’s disease. As a result, conclusions related to EOAD should be interpreted cautiously and considered hypothesis-generating rather than definitive evidence of disease-specific mechanisms.

Finally, the genomic datasets used in this study predominantly represent populations of European ancestry. Therefore, the applicability of these findings to other populations, including those in Latin America, should be interpreted with caution.

This approach aims to generate new hypotheses and identify promising candidate genes and regulatory mechanisms supported by human genetic evidence. Importantly, our findings—particularly *GSE1* as a key regulatory signal in EOAD and *APH1B/CEP63* as broadly regulated genes in LOAD—represent candidates that require further functional validation rather than confirmed disease mechanisms. Moving forward, we emphasize that testing these regulatory effects in relevant cellular or animal models will be crucial to understanding their actual biological impact.

Future studies integrating cell-type-specific transcriptomic data, age-stratified cohorts, and independent replication datasets will be essential to refine and validate these findings.

## 6. Conclusions

This integrative analysis highlights regulatory signals that may contribute to Alzheimer’s disease-related genetic risk through gene expression mechanisms. By combining GWAS summary statistics with transcriptomic data, the study identifies candidate loci and genes that warrant further functional investigation.

Although the findings are consistent with previously reported pathways implicated in Alzheimer’s disease, their relevance to early-onset Alzheimer’s disease remains provisional. The results should therefore be interpreted as exploratory and hypothesis-generating, rather than as evidence of EOAD-specific mechanisms. Furthermore, while this study highlights distinct regulatory architectures between EOAD and LOAD, these conclusions are based on bulk tissue eQTL data and therefore reflect tissue-level regulatory signals. Integrating cell-type-resolved transcriptomic and eQTL datasets will be necessary in future studies to refine cellular specificity and establish the causal relevance of the regulatory mechanisms identified here.

Overall, this work highlights the value of integrative genomic approaches for prioritizing candidate genes and pathways, while emphasizing the need for future studies incorporating age-stratified cohorts, cell-type-specific data, and functional validation to clarify disease mechanisms across different forms of Alzheimer’s disease.

## Figures and Tables

**Table 1:** Chromosomal distribution of variants associated with EOAD and LOAD. Only the 10 variants with the highest statistical significance in each group are included.

Chr.	EOAD			LOAD		
	No. of Variants	Top Significant Variant(s) rsID (*Gene*)	*p*	No. of Variants	Top Significant Variant(s) rsID (Gene)	*p*
1	1			17	rs679515 (*CR1-AS1*, *CR1*)	2.0 × 10^−25^
2	7	rs11903348 (*RPL38P2*, *LINC01851*) rs9678754 (*LINC01826*, *LINC01823*)	7.0 × 10^−7^ 2.0 × 10^−6^	22	rs4663105 (*NIFKP9*, *BIN1*)	4.0 × 10^−58^
3	0			11		
4	0			8		
5	3	rs75661618 (U4, ADAMTS2)	2.0 × 10^−6^	14		
6	1	rs1963159 (*NQO2*)	2.0 × 10^−6^	18	rs187370608 (*UNC5CL*, *LRFN2*) rs75932628 (*TREM2*)	1.0 × 10^−25^ 5.0 × 10^−24^
7				19		
8	2			13		
9	2			10		
10	1	rs41301613 (*ZNF22-AS1*)	2.0 × 10^−6^	6		
11	1	rs111785670 (*MIR100HG*)	2.0 × 10^−6^	18	rs1582763 (*MS4A4A*) rs561655 (*PICALM*, *RNU6-560P*)	3.0 × 10^−33^ 1.0 × 10^−26^
12	2			14		
13	0			8		
14	1			6		
15	0			11		
16	2	rs55889290 (*CES5A*)	4.0 × 10^−7^	12		
17	4	rs62061022 (*PIMREG*) rs33928449 (*EXOC7*) rs12942395 (*ACTG1*, *LINC01971*)	6.0 × 10^−17^ 1.0 × 10^−6^ 2.0 × 10^−6^	14		
18	1			3		
19	1	rs769449 (*APOE*)	3.0 × 10^−15^	21	rs429358(*APOE*) rs7412 (*APOE*) rs10119 (*TOMM40*) rs157591 (*APOC1*, *APOC1P1*)	1.0 × 10^−300^ 3.0 × 10^−105^ 2.0 × 10^−57^ 4.0 × 10^−29^
20	0			5		
21	0			2		
22	0			3		

**Table 2:** Distribution of GWAS variants by type of functional consequence in EOAD and LOAD.

Most_Severe_Consequence	EOAD	LOAD
3 prime UTR variant	0	6
Downstream gene variant	0	13
Intergenic variant	14	78
Intron variant	15	111
Missense variant	0	18
Non-coding transcript exon variant	2	7
Regulatory region variant	1	2
Synonymous variant	0	3
Upstream gene variant	0	17

**Table 3:** eQTL-regulated genes and their tissue-specific distribution in EOAD and LOAD.

Gene	# SNP	Tissue-Specific	Disease
*GSE1*	1 (rs58675609)	Brain_Frontal_Cortex_BA9	EOAD
*PMS2P1*	1 (rs7384878)	Brain_Amygdala	LOAD
*CEP63*	1 (rs142076474)	Brain_Anterior_cingulate_	LOAD
*CEP63*	1 (rs142076474)	Brain_Caudate_basal_ganglia	LOAD
*CEP63* *FUT2*, *MAMSTR*	1 (rs142076474) 1 (rs2452170)	Brain_Cerebellar_Hemisphere	LOAD
*CEP63*	1 (rs142076474)	Brain_Cerebellum	LOAD
*CR1-AS1*, *CR1*	1 (rs679515)	Brain_Cortex	LOAD
*APH1B* *APOE* *KLHL36*, *USP10*	1 (rs117618017) 1(rs7412) 1 (rs12716755)	Brain_Frontal_Cortex_BA9	LOAD
*CEP63* *HAVCR2*	1 (rs142076474) 1 (rs6891966)	Brain_Hypothalamus	LOAD
*ABCA7*	1 (rs12151021)	Brain_Nucleus_accumbens_basal_ganglia	LOAD
*APH1B* *APOE*	1 (rs117618017) 1(rs7412)	Brain_Putamen_basal_ganglia	LOAD
*APH1B* *HAVCR2*	1 (rs117618017) 1 (rs6891966)	Whole_Blood	LOAD

## Data Availability

The data supporting the findings of this study are available in the Supplementary Materials. An Excel file containing the data used for the analyses has been provided as part of the manuscript submission.

## Supplementary Materials

The following supporting information can be downloaded at: https://www.mdpi.com/article/10.3390/genes17020186/s1, detailed eQTL results for all analyzed tissues are provided in Supplementary Materials (Excel file).
